# Chicago Medical Society

**Published:** 1884-12-20

**Authors:** 


### CHICAGO MEDICAL SOCIETY.

At the regular meeting of November 10, ultimo, the president, Dr. D. A. K. Steele, presided.

We presented to our readers, in the October number of the RECORD, a series of resolutions submitted by Dr. L. H. Montgomery, the secretary of the Society, and herewith publish the committee's special report, under the head of National Sanitation, as being carefully prepared and appropriate, as follows :

MR. PRESIDENT : The committee appointed at the meeting of this Society, September 15, 1884, to consider and report upon a series of resolutions presented by Dr. Liston H. Montgomery, having reference to national sanitary matters, respectfully report the following preamble and resolutions as suitable to be adopted :

Whereas, Experience has firmly established the fact that the ravages of certain infectious and contagious diseases may be, in good measure, prevented, restricted or arrested by the enforcement of suitable sanitary regulations ; and

Whereas, The United States is constantly exposed to the importation of disease from foreign countries, and, because of the facility and rapidity of inter-State transit, to the rapid spread of infection once finding lodgment upon our borders ; and

Whereas, This exposure, because of the prevalence of cholera in Europe, is just now unusually great ; and

Whereas, The facts are, that matters of sanitation are, in some of the States of this Union entirely neglected, while in others they are simply taken cognizance of by the appointment of boards of health in their functions advisory only, and unclothed with powers of authoritative action ; and

Whereas, Either of these State boards of health, as now constituted, may prove derelict or inefficient in its duties, or act without concert with, or even in antagonism to, the boards of other States ; and

Whereas, The exigencies occasioned by the appearance of violent epidemics demand organized means for the prompt recognition of the outbreak of disease, and vested authority, limited in its area by the boundaries of the country only, to take such immediate steps in matters of protection as vaccination, isolation, quarantine, etc., as experience has taught to be useful; and

Whereas, No National authority in sanitary matters now exists; therefore,

Resolved, That it is the judgment of the Chicago Medical Society that the sanitary interests of the United States demand the establishment of a permanent national health authority, which shall have for its main functions the detection of pestilential and epidemic diseases, and the enforcement, where necessary, of sanitary regulations tending to prevent, to abate, or to suppress them.

Resolved, That, as a step toward the consummation of the ideas suggested in the foregoing resolution, a committee of three be appointed by this Society to collate facts tending to show the usefulness and necessity of a national sanitary organization, to compile the same in such form as may be available for disseminating information upon, and creating an interest in, national sanitary legislation.

Resolved, That said committee be empowered and instructed to urge the importance of national legislation upon the attention of the Congressional delegation from Illinois, and fittingly to present the subject to the representatives of the people in both houses of Congress.

All of which is respectfully submitted.

O. C. DEWOLF, Chairman,

R. E. STARKWEATHER,

L. H. MONTGOMERY,

JOHN BARTLETT,

J. H. ETHERIDGE,

A. R. JACKSON,

J. H. HOLLISTER,

Committee.

The suggestions embodied in the above were unanimously adopted, and the new committee was appointed.

